# Items

**Published:** 1881-04

**Authors:** 


### ITEMS.

HOMŒOPATHIC ACHIEVEMENTS.—A Homoeopath, of the name of O. B. Bird, is collecting an epitome of homœopathic achievements. The first "achievement" which we see recorded is this: "Diphtheria, 1856, Dr. P——, Washington, 100 cases, 3 deaths. Under allopathic treatment two-thirds of all cases died. Franklin County, N. Y., Dr. H——, 1860-62, treated 1,000 cases of diphtheria, lost 8 per cent.; best allopath lost 25 per cent, Lowell, Mass., severe epidemic dysentary: allopaths lost 10 per cent., Drs. H—— and S——, homepaths, lost $1\frac{1}{2}$ per cent.," etc. And yet homoeopathy is losing ground.—*Medical Record.*

Will "*Medical Record*" kindly furnish the public with some evidence that "homœopathy is losing ground?" Does it refer to burying *ground*?

HOSPITAL FOR INCURABLES.—Henry C. Gibson, Esq., a well known citizen of Philadelphia, has made a donation of $50,000 to the Incurable Ward of the Hospital of the University of Pennsylvania. A ball on a large scale will soon be given by the leaders of Philadelphia society for the purpose of raising additional funds for this laudible object.—*Medical Record.*

The allopaths *need* large, airy "incurable wards" for there it is that most of their patients live.

"MEDICAL HERECIES."—In his review of Dr. Symthe's book on Medical Heresies, Dr. James wrote: "When an allopathic doctor finds his patient going over to the new 'heresy' he will simply place in his hands a copy of this book and thereby change his intention." An allopathic journalist thus confirms Dr. James' prophecy; speaking of the volume he says: "It exposes the absurdity of its fundamental doctrine of similars, and the impossibility of its numerous triturations and dilutions; and, finally shows how, at the present time, many of those who call themselves homœopaths are teaching and practicing something very much like what regular physicians inculcate. If a copy of this very readable, clear, and convincing book could be placed in the hands of every intelligent person who practices or supports homœopathy, they would see how egregiously they are being duped."—ED.

*Nullius addictus in verba magistri jurare* is the declaration of many unbelievers in Hahnemann's "Organon," yet these same persons secretly pin their faith on the theories and advice of current authors!!

We received the "extra" of the St. Louis *Clinical Review*, containing the speeches and poems delivered, Oct. 10th, 1880, in honor of Constantine Hering. The St. Louis physicians do themselves honor in thus paying tribute to the great homoeopath. And the Philadelphia fraternity are yet childishly quarrelling as to whether they have had a meeting or not!

At its meeting of Dec. 8th, 1880, the "*Societe Hahnemannienne Federative*" endorsed the "Declaration of Homoeopathic Principles."

